# From grass to grace: How SLMTA revolutionised the Bamenda Regional Hospital Laboratory in Cameroon

**DOI:** 10.4102/ajlm.v3i2.203

**Published:** 2014-11-03

**Authors:** Siyem C. Nkwawir, Nakeli N. Batumani, Talkmore Maruta, Charles N. Awasom

**Affiliations:** 1 Bamenda Regional Hospital Laboratory, Cameroon; 2 African Society for Laboratory Medicine, Ethiopia

## Abstract

**Background:**

Public health laboratories form the foundation on which today’s clinical laboratory practice in Cameroon is built. The advent of the Strengthening Laboratory Management Toward Accreditation (SLMTA) programme in 2009 empowered the Bamenda Regional Hospital Laboratory (BRHL) to improve its working culture, practices and management.

**Objectives:**

To evaluate the results of SLMTA implementation at BRHL and discuss lessons learned.

**Method:**

In 2010, the SLMTA programme was rolled out in Cameroon to improve laboratory quality management systems in five laboratories, including BRHL. Three workshops were conducted (the first centralised, the remaining two on-site at each laboratory) and improvement projects were implemented after each workshop with the assistance of mentors. Audits were used in order to evaluate performance and to identify areas for further improvement.

**Results:**

BRHL had the lowest score (18%) amongst the cohort at the baseline audit and the highest (81%) at the official Stepwise Laboratory Quality Improvement Process Towards Accreditation (SLIPTA) audit conducted in August 2013 by the African Society for Laboratory Medicine. Improvements were observed in each of the 12 Quality System Essentials; improvement was especially noteworthy in the areas of facilities and safety, and purchasing and inventory. Staff investment and pride in the quality of laboratory services increased.

**Conclusion:**

BRHL’s remarkable improvement was achieved with a combination of SLMTA training activities, intensive on-site mentorship and the collective focus of all laboratory staff. The experience at Bamenda Hospital illustrates what can be achieved when a laboratory successfully harnesses the energy of its staff and implements changes to improve the quality of services in a transformation taking them from grass to grace.

## Introduction

The need to improve health outcomes regionally has led health systems and governments in Africa toward evidence-based medicine, which requires access to high-quality information such as laboratory tests for decision making.^1^ Given the emphasis on reliable data, increased attention has been focused on medical laboratories and their quality of services. In 2008, at a meeting in Yaoundé, Cameroon, organised by the World Health Organization’s Regional Office for Africa (WHO AFRO), leaders from several African countries recognised the critical role of public health laboratories in disease control and prevention and resolved to strengthen public health laboratories throughout the region.^2^ In the following year, WHO AFRO launched a stepwise laboratory accreditation preparation scheme, later called the Stepwise Laboratory Quality Improvement Process Towards Accreditation (SLIPTA).^3^ SLIPTA provides a framework for benchmarking progress using an audit checklist based on International Organization for Standardization (ISO) 15189:2007 requirements. At the same meeting, the Strengthening Laboratory Management Toward Accreditation (SLMTA) programme was launched in order to help support laboratory quality improvement. SLMTA is a laboratory management training programme which follows a multi-workshop implementation model^4^ and supports the development of the 12 Quality System Essentials (QSEs) of the Clinical and Laboratory Standards Institute (CLSI) Laboratory Quality Management System Guidelines.^5^

Cameroon is a Central African country with a population of approximately 20 million people living in 10 regions.^6^ Bamenda is the headquarters of the Northwest region, which has a population of approximately 1.8 million.^6^ Bamenda Regional Hospital, a 400-bed public health hospital inaugurated in 1956, serves as the referral hospital for the 19 district hospitals of the Northwest region.

The field of laboratory medicine in Cameroon has developed in several phases, starting with the paramedic ‘Medical Field Unit’ introduced before independence in 1961. These laboratory medicine professionals received minimal training; their primary role was field identification of contagious diseases such as varicella, measles, yaws, smallpox, leprosy and tuberculosis. In most cases, they used basic identification techniques in mobile units.^7^ In 1968, a decree by the Ministry of Health led to the establishment of an organisational structure within public hospitals and health units.^8^ The hospital’s chief pharmacist became the laboratory head, under the supervision of the hospital director or a medical doctor who had specialised qualifications in microbiology, biochemistry, haematology or serology. At the time, laboratory services were either free or offered at minimal cost to the patient.^8^ Two decades later, in 1990, a subsequent decree established organisational structures within private clinical laboratories.^9^

These statutes were designed to ensure the widespread provision of affordable laboratory services. They did not, however, address the quality of those services. In Cameroon, the public health sector, in particular, has been characterised by a *laissez-faire* approach, with little concern for client satisfaction, limited specialised skills and mismanagement of both human and financial resources.^10,11,12^

In an effort to strengthen laboratory management so as to achieve rapid laboratory quality improvement, the SLMTA programme was launched in Cameroon in October 2010. The Bamenda Regional Hospital Laboratory (BRHL) was selected as one of five laboratories in the initial SLMTA cohort. Laboratory personnel and other hospital staff participated in a series of training activities and quality improvement projects in an effort to enhance laboratory management, provide reliable patient services and create a lasting culture of quality. This article discusses SLMTA implementation at BRHL, as well as results achieved and lessons learned.

## Research methods and design

### Phase 1: Preparation

The US Centers for Disease Control and Prevention (CDC) served as the sponsor for SLMTA implementation in Cameroon, with Global Health Systems Solutions (GHSS) as the implementing partner. Five laboratories (Yaoundé Central Hospital Laboratory, BRHL, Buea Regional Hospital Laboratory, Douala Laquintinie Hospital Laboratory and Laboratoire d’Analyse Medicales du Centre Yaoundé) were selected based on commitment from hospital laboratory management, appointment of a quality officer and the availability of participants who would maintain the same job responsibilities through the duration of the programme.^4^

Prior to SLMTA implementation, local capacity for auditors, trainers, and mentors was developed. In 2009, seven Cameroonian auditors were trained to evaluate laboratory quality management systems (QMS). Five Cameroonian laboratory personnel completed the SLMTA training-of-trainers course. In 2010, an experienced mentor and instructor from the Clinton Health Access Initiative (CHAI), Lesotho, trained 11 Cameroonian laboratory mentors. Along with training on international standards, mentors were taught to build a sustainable culture of quality by transferring their knowledge and expertise to bench technologists.

During the planning stages, CDC and GHSS organised an advocacy meeting with hospital directors and laboratory managers of the five laboratories to ensure their buy-in and commitment to laboratory improvement. BRHL further engaged hospital management for support in implementing QMS, through meetings and active involvement in the laboratory improvement process. Several hospital managers were also invited to attend the on-site SLMTA trainings, which helped encourage their support and collaboration. Additionally, all hospital staff members were briefed on the laboratory improvement efforts.

### Phase 2: SLMTA programme implementation

The 12 SLMTA modules were taught in a series of three workshops, each lasting four days, over a period of eight months (Table 1). The five in-country SLMTA trainers facilitated the programme.

**TABLE 1:** SLMTA Workshop Topics, Bamenda Regional Hospital Laboratory.

Schedule	Location	Date	Modules taught
Workshop 1	Mutengene, Cameroon	October 2010	Introduction Cross-cutting Productivity management Quality assurance Documents and records management
Workshop 2	On-site at BRHL	February 2011	Work area management Inventory management Procurement management Routine and/or preventive maintenance of equipment
Workshop 3	On-site at BRHL	June 2011	Specimen collection and processing Laboratory testing Test result reporting

SLMTA, Strengthening Laboratory Management Toward Accreditation; BRHL, Bamenda Regional Hospital Laboratory.

The first workshop was conducted in Mutengene, Cameroon (October 2010) for staff from the five targeted laboratories, including four staff members from BRHL. This workshop focused on the cross-cutting, productivity management, quality assurance, and documents and records management modules. Following the first workshop, the SLMTA coordinators and trainers decided that it would be more effective and practical to hold the remaining two SLMTA training workshops at each of the enrolled laboratories so as to allow for more personnel to be trained. Therefore, BRHL’s second and third workshops were conducted on-site at the Bamenda Regional Hospital. To maximise impact, 17 staff members attended the training, including the heads of the blood bank, reception and customer service, haematology, biochemistry, microbiology, serology and parasitology, as well as the safety officer, equipment officer, quality officer, laboratory director and hospital director. The 13 participants who missed the first centralised workshop were given materials to review.

After each workshop, participants implemented improvement projects based on areas of weakness in the laboratory. All laboratory staff were involved in the improvement projects, including those who did not attend the training.

Following the training, GHSS mentors were deployed to the five targeted laboratories, spending extended periods of time on site. The structured peer-to-peer, side-by-side mentorship approach was used to impart knowledge and expertise from the mentor to the laboratory staff following set guidelines.^13,14^ Two additional mentors were assigned to BRHL, alternating supervisory visits and working with all staff members to implement a change in culture and address gaps. ISO 15189, the SLIPTA checklist and the SLMTA toolkit were the primary tools used for follow-up. The mentors helped staff members address areas requiring improvement by examining the SLMTA toolkit, developing improvement projects, repeating activities from the training as needed, and then measuring improvement achieved using the checklist.

### Phase 3: Evaluation

A series of audits using the SLIPTA checklist were conducted and scores were calculated for each of the 12 QSEs so as to pinpoint problem areas and guide the development of improvement projects. Overall scores were calculated as a percentage of the maximum possible points. To benchmark progress, these scores were also broken into ‘star’ levels, with zero stars corresponding to < 55%, one star 55% – 64%, two stars 65% – 74%, three stars 75% – 84%, four stars 85% – 94% and five stars 95% – 100%.

The baseline audit was conducted 11 months prior to SLMTA implementation (November 2009). Interim audits were conducted at various points (October 2010 before workshop 1; June 2011 before workshop 2; December 2011 after workshop 3; and February 2012). The exit audit was conducted in September 2012. These audits were performed by locally-trained auditors who were neither SLMTA trainers nor mentors for the BRHL staff. Finally, in August 2013, the African Society for Laboratory Medicine (ASLM) conducted an official SLIPTA audit.

## Results

**FIGURE 1:**
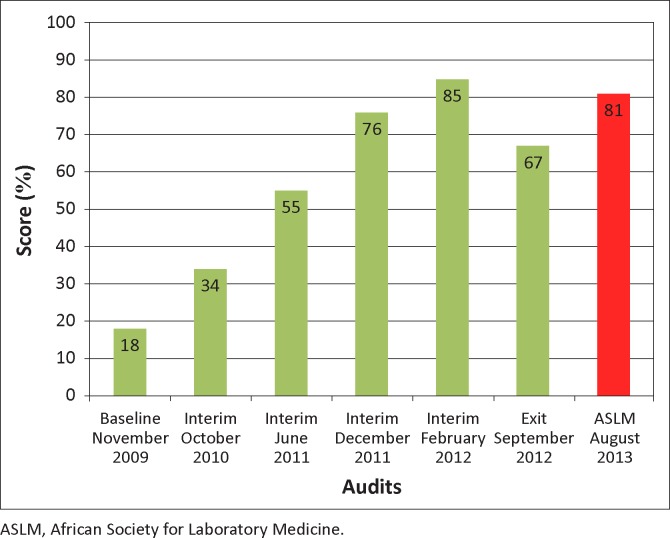
Progress of the Bamenda Regional Hospital Laboratory in quality management systems as measured by the Stepwise Laboratory Quality Improvement Process Towards Accreditation (SLIPTA) checklist from November 2009 to August 2013.

BRHL’s performance progressed steadily from 18% (zero stars) at baseline, where it was ranked last amongst the laboratories in its cohort, to 85% (four stars) in an interim audit eight months after completion of the three workshops, but then dropped to 67% (two stars) at the exit audit seven months later (Figure 1). At the official ASLM audit, the laboratory scored 81% (three stars), and was ranked first amongst the four laboratories audited, with the others scoring 60% – 72%.

**FIGURE 2:**
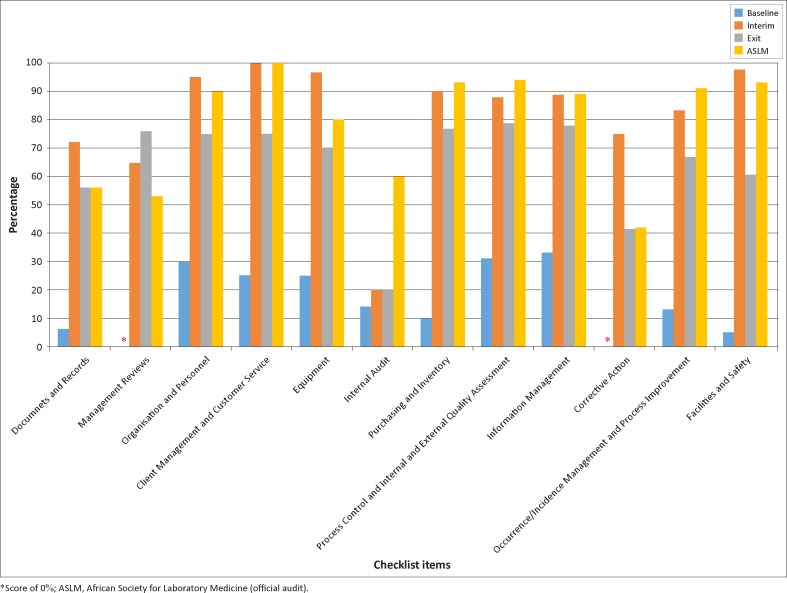
Performance of the Bamenda Regional Hospital Laboratory (%) across the 12 Quality System Essentials at baseline, last interim, exit and official ASLM audit.

Improvement was observed in each of the 12 QSEs measured in the SLIPTA checklist (Figure 2). From baseline to the last interim audit in February 2012, the most substantial improvements were in facilities and safety (93 percentage points), and purchasing and inventory (80 percentage points), whilst the least improved area was internal audit (6 percentage points). From the last interim to the exit audit, there were noted decreases in three areas: facilities and safety (37 percentage points), corrective action (33 percentage points), and equipment (27 percentage points). At the official ASLM audit, the strongest areas were client management and customer service (100%), process control and internal and external quality assessment (94%), purchasing and inventory (93%) and facilities and safety (93%); whilst the weakest areas were corrective action (42%), management reviews (53%) and documents and records (56%).

Improvement projects were selected and implemented after each SLMTA workshop (Table 2). After each audit, the summary of non-conformities and recommendations was reviewed by the quality team and additional improvement projects were assigned, depending on what was feasible at the time, available resources and the seriousness of the non-conformity.

**TABLE 2:** A summary of improvement projects, Bamenda Regional Hospital Laboratory, Cameroon.

Problem	Improvement project	Period of implementation
Lack of documented procedures	Establish Standard Operating Procedures (SOP).	After SLMTA workshop 1
Missing specimens	Improve specimen management (labelling, logging, analysis and proper disposal).	After SLMTA workshop 1
Unsafe environment	Tile the floors and work stations; improve the use of personal protective equipment; establish a safety manual; extend building and improve floor plan; establish triple packaging; install proper signage.	After SLMTA workshop 2
Frequent stock outs	Improve inventory and stock management (reagent logs, stock cards, invoice recording, assessment of supplies and suppliers).	After SLMTA workshop 2
Congestion and clutter	Establish equipment identification and management (labelling, removal of obsolete equipment).	After SLMTA workshop 2
Poor client satisfaction	Establish tools for customer satisfaction survey (suggestion box, survey forms, feedback from clinicians through meetings); establish client handbook.	After SLMTA workshop 3
Poor non-conformity management	Establish a system to manage non-conformities including how to capture them; establish a team to investigate and implement corrective action.	After SLMTA workshop 3
Poor quality management	Improve internal quality control documentation (logs, registers); establish quality indicators.	After SLMTA workshop 3
Poor reviewing system	Assign supervisory personnel in charge of biosafety, quality, and equipment.	After first interim audit
Poor document control	Establish a document control system (manage present and archival documents).	After exit audit

SLMTA, Strengthening Laboratory Management Toward Accreditation.

## Discussion

BRHL’s substantial improvement was achieved by means of a combination of training activities, intensive on-site mentorship and collective focus of all laboratory staff. The experience at BRHL illustrates what can be achieved when a laboratory successfully harnesses the energy of its staff and implements changes in order to improve the quality of services.

Engaging the management of Bamenda Hospital was key to ensuring financial support and sufficient human resources for the quality improvement process. The hospital director attended several of the SLMTA trainings, engaged directly with the mentor on quality management issues and increased the frequency of his visits to the laboratory. With management buy-in, the laboratory implemented infrastructure renovations and gained financial support for the purchase of reagents.

Since SLMTA completion, meetings between clinicians and laboratory staff have continued on a regular basis so as to enable laboratory staff to inform and consult with those they now see as critical clients and partners. Because of these meetings, corrective actions have been easier to implement, customer satisfaction surveys have been facilitated and trust has been built between these users and providers of laboratory services. For BRHL patients, the improvements have resulted in better service delivery because of procedure standardisation, fewer recalls of specimens, a safer environment, more reliability of testing and the ability to provide feedback on services offered.

Cameroon was the first country to decentralise SLMTA training,^15^ conducting the second and third workshops on-site at each enrolled laboratory. Moving the SLMTA trainings to the facility allowed more staff to be trained and helped encourage universal participation and enthusiasm amongst all staff. Whilst there was resistance to change in the beginning, by the third training there was a strong zeal to increase the laboratory’s star rating, as can be seen in the exit audit results. Staff members became optimistic as the scores started to rise; their determination to improve the laboratory’s ratings was evidenced by their willingness to work extra hours, including weekends and public holidays. One young female laboratorian in the Microbiology department said, ‘SLMTA has made us more ambitious. I now look forward to progressing in this profession and being able to help people of my community’.

Initially, the laboratory implemented what was possible at the time, rather than waiting for the ideal situation to arise. The quick successes of initial improvement projects bolstered staff confidence and inspired the desire for success, helping the laboratory sustain motivation for the more difficult projects. These successes also helped garner additional technical and financial assistance for further changes. Whilst some improvement projects dealt with day-to-day activities, others sought to address larger challenges and attain more long-term goals. One noteworthy improvement project was to reorganise the reception area, which resulted in the creation of multiple reception sites, increased confidentiality and reduced turnaround time for test results. Better organisation is a common theme in SLMTA training; one elderly female participant noted, ‘It has even extended to my home. Now all my cupboards in the kitchen are labelled. All junk I removed’. Laboratory staff members have reported a change in the culture of the laboratory. One commented that ‘our patients have ceased to be patients; they are now our clients’. The hospital director agreed, saying, ‘If I travel, I am now confident that work still continues, even in my absence’. He even took a leading role in directing improvement projects, personally supervising the closing of gaps in preparation for the official ASLM audit. He met twice daily with quality team members for a period of three weeks; daily tasks were identified every morning and achievements and bottlenecks were reported every evening.

A full-time mentorship approach allowed the on-site mentor to participate in daily activities in the laboratory and provide targeted training and guidance regarding the implementation of various aspects of the QMS. The SLMTA-trained mentors helped build a working culture that emphasised quality and a sustainable QMS. Their continued presence over time helped them understand the local culture and practices, enabling them to design a tailored approach to assisting the laboratory in quality improvements. By working side-by-side with staff as peer coaches and role models, the mentors gained acceptance by laboratory personnel and accelerated implementation of quality requirements.

The laboratory's performance increased steadily through SLMTA implementation, but the dramatic drop in the exit audit score 15 months after the last workshop highlights the importance of focusing on sustainability. Staff had viewed the attainment of improved scores as the end of the struggle, rather than as part of an ongoing process of continuous quality improvement, requiring the maintenance of a new culture of quality. For example, client management and customer satisfaction, purchasing and inventory, process control and occurrence management all improved substantially from baseline to the last interim audit, but then declined once the SLMTA programme had ended and focus was lost. After the drop in score at the exit audit, the laboratory staff redoubled their efforts and results for these areas increased substantially once again by the official ASLM audit. One area that did not improve after the exit audit was documents and records. Whilst documentation improved from 6% at baseline to 72% at the last interim audit, it then dropped to 56% at exit where it remained at the official audit. The noted improvement resulted from the development and authorisation of many new policies and procedures, including a quality manual. However, by the time of the exit audit, the laboratory had begun to experience new challenges associated with the expanded documentation structure; the laboratory was overwhelmed by the number of documents that needed review and the ongoing maintenance required to retrieve documents, archive obsolete documents and communicate new changes. This experience highlights the need to plan ahead and to consider not only the development but also the maintenance of new systems.

A new quality culture is growing at BRHL as staff have embraced the need for continuous improvement and excellence in patient care. However, there is now a critical need to develop components outside the reach of the laboratory’s control: updated national guidelines for policies and procedures, professional boards for licensure, an up-to-date electronic database for proper document control and management, a hospital improvement programme to keep pace with improvements in the laboratory, regular audits of the QMS and qualified service engineers. Though the ultimate goals of reaching five stars and international accreditation have not yet been realised at BRHL, the measurable improvement to date is a catalyst for greater achievements as the laboratory continues its journey from grass to grace.
